# Evidence for an inhibitory-control theory of the reasoning brain

**DOI:** 10.3389/fnhum.2015.00148

**Published:** 2015-03-23

**Authors:** Olivier Houdé, Grégoire Borst

**Affiliations:** ^1^CNRS Unit 8240, Laboratory for the Psychology of Child Development and Education, Alliance for Higher Education and Research Sorbonne-Paris-Cité, Paris Descartes UniversityParis, France; ^2^Institut Universitaire de FranceParis, France

**Keywords:** inhibitory control, reasoning, heuristics/algorithm, developmental cognitive neuroscience, Piaget

## Abstract

In this article, we first describe our general inhibitory-control theory and, then, we describe how we have tested its specific hypotheses on reasoning with brain imaging techniques in adults and children. The innovative part of this perspective lies in its attempt to come up with a brain-based synthesis of Jean Piaget’s theory on logical algorithms and Daniel Kahneman’s theory on intuitive heuristics.

Based on the numerous scientific data garnered in children of all ages, Jean Piaget (Piaget, 1983) proposed a seminal model of cognitive development according to which children’s cognitive abilities developed through four different stages from the sensorimotor stage (from birth to 2 years of age) to the formal operational stage (starting at 12 years of age). Between two and 7 years of age (the so-call preoperational stage), Piaget assumed that children were mainly illogical in comparison to adults. Importantly, during the concrete operational stage, between 7 and 12 years of age, children start to reason logically in several logico-mathematical domains (e.g., number, categorization…). Finally, after 12 years of age, children’s reasoning is not limited to concrete objects but can be applied to abstract propositions.

## Inhibitory-Control Theory as an Alternative to Piaget’s Theory

In fact, Piaget underestimated the rich precocious logical knowledge already present in infants and young children, and he overestimated the logical abilities of older children, adolescents and adults, who commit systematic errors even in very simple logical tasks (Houdé, 2000; Kahneman, 2011). These logical errors usually occur when older children, adolescents and adults rely on prepotent responses, illogical intuitions, or misleading strategies (such as heuristics) rather than on logical algorithms. Importantly, the ability to overcome those errors is directly related to the ability to inhibit these intuitive forms of thinking (Houdé, 2000; Kahneman, 2011; Houdé and Borst, 2014). Consequently, today the discrete Piagetian stages theory is replaced by an approach of cognitive development which is analogous to overlapping waves within a non-linear dynamic system (Siegler, 1999). In such a system, at any point in time and at any age, different strategies with different degrees of complexity and sophistication might be in conflict in the brain. According to this theoretical framework, the progressive ability of the prefrontal cortex to inhibit irrelevant or misleading strategies to activate the most logical one sustains the conceptual development of children and the shift from one Piagetian stage to the next (Houdé and Borst, 2014). This constitutes the central assumption of our new neo-Piagetian theory of reasoning development.

During cognitive development, children and adults have to choose, depending on the context, between two types of strategies or multiple levels of “thinking fast and slow” (Kahneman, 2011). Typically, individuals can either solve problems using heuristics (i.e., intuitions) or logico-mathematical algorithms. On the one hand, heuristics are typically defined as strategies that are effortless, rapid, often global or holistic which constitute the most adaptive response in most situations but sometimes they are misleading especially in situations in which they compete with logical algorithms. Algorithms, on the other hand, are slow, analytical and cognitively costly strategies but they always provide the correct solution independently of the context. In most contexts, children and adults spontaneously rely on heuristics. However, choosing heuristics over algorithms does not mean that children and adults are irrational *per se* (Houdé, 2000) or “happy fools” (De Neys et al., 2013). A “presumption of rationality” is sometimes the best assessment.

## Brain Imaging of Reasoning-Bias Inhibition in Adults: The Example of Deductive Logic

As opposed to Piaget’s theory, which assumed that children reached a logical stage of reasoning at 12 years of age (i.e., formal operational stage), a number of studies have now provided converging evidence that adolescents and adults continue to make errors in simple deductive reasoning tasks (see e.g., Evans, 1998, 2003; Houdé, 2000). For instance, in the perceptual matching bias task designed by Evans (Evans, 1998), the vast majority of participants choose a red square on the left of a yellow circle to falsify the following rule: “*if there is not a red square on the left, then there is a yellow circle on the right*”. Evans attributed this error of logic to a perceptual matching bias (or heuristic) according to which participants choose the two geometrical shapes mentioned in the rules because a negation is present in the antecedent rather than using the logical truth table (in this case the algorithm). By using the logical truth table, participants would chose two geometrical shapes (e.g., a blue diamond to the left of a green square) validating a true antecedent (i.e., not a red square) and a false consequent (i.e., not a yellow circle). Critically, in order to avoid systematic logical errors in this context, participants must resist (or inhibit) the perceptual matching bias (i.e., red square on the left of a yellow circle) to activate the logical algorithm.

According to our “presumption of rationality” analysis, participants’ difficulty in solving this if-then logical problem is not related to the difficulty of the deductive reasoning *per se* but to the difficulty to exert inhibitory control over the misleading heuristic (i.e., the perceptual matching bias). To provide evidence for the role of inhibitory control in overcoming deductive reasoning errors, we contrasted the effect of two types of training on the ability to perform deductive reasoning tasks. In one condition, participants were trained to inhibit the perceptual matching bias. In the other condition, participants received training focusing on explaining the underlying logic of the task. Importantly, participants were trained on a different deductive task (i.e., the Wason task, Wason, 1968) than the one performed pre- and post-training (i.e., the perceptual matching bias task, Evans, 1998). The effects of the two types of training were compared to a test-retest control condition in which participants simply performed the perceptual matching task two times. Participants who were trained to inhibit the perceptual matching heuristic were the only ones who succeeded to overcome their deductive reasoning errors. This finding suggests that logical reasoning errors are not due to a lack of logic (or experience) but to a default to inhibit a misleading heuristic. In a follow-up PET (positron emission tomography) imaging study in which we compared the cerebral activation before and after the participants were trained in inhibiting the perceptual matching bias, we observed that the brain activation shifted from the posterior perceptual regions pre-training to prefrontal executive regions post-training. This is the first micro-longitudinal neuroimaging study of deductive reasoning and it provides the first evidence that inhibitory control was critical to reason logically.

Note that this brain imaging study on reasoning errors correction was conducted on a sample of only eight participants but the strength of these results stem from the fact that the participants were their own controls in the pre-post training comparison. Such intra-individual design is scarce in brain imaging of reasoning. Indeed Fuster (Fuster, 2003), noted about our results that “the exercise of logical reasoning seems to overcome (or to inhibit) the biasing influences from the posterior cortex and to lend to prefrontal cortex the effective control of the reasoning task” (p. 231). More specifically, with respect to our results in the prefrontal cortex, we observed a left-middle-frontal gyrus activation which was likely to reflect the logical manipulation of the algorithm in working memory, and a left-inferior-frontal gyrus activation, which was likely to reflect inhibition of the reasoning bias (or heuristic) and self-regulatory inner speech (Broca’s area).

In this brain imaging study, the training condition that focused on the inhibition of the misleading heuristic comprised not only cognitive but also emotional executive warnings that were not incorporated in the training condition focusing on explaining the underlying logic of the deductive problem. By directly contrasting the cerebral activity elicited by the two types of training, we found greater activity (i.e., the rCBF: regional cerebral blood flow) following inhibitory control training in the right ventromedial prefrontal cortex (Houdé et al., 2001), which is a paralimbic emotional area (Mesulam, 2000) known to be involved in getting the mind on the “logical track” and avoiding decision-making errors (Damasio et al., 1994; Damasio and Carvalho, 2013). We speculate that the right ventromedial prefrontal cortex could serve as an internal warning/self-feeling device to correct errors during deductive reasoning. Converging data on the link between emotion, conflict detection and inhibition were reported by Spiess et al. (2007) and De Neys et al. (2010).

After these two pioneer brain imaging studies on *if-then* rules (Houdé et al., 2000, 2001), a set of new studies were published during the past decade on deductive reasoning (e.g., Noveck et al., 2004; Prado and Noveck, 2007; for reviews see Goel, 2007; Prado et al., 2011). Noveck et al. (2004) studied the underlying brain network engaged in deductive reasoning on abstract contents and found that a left lateralized parietal-frontal network supported the if-then (or conditional) reasoning. Importantly, the activation within this network increased as the reasoning became more complex. As noted by Noveck et al. (2004), a critical difference between their study and the two neuroimaging studies we conducted was that solving Evans’s problem required a counterintuitive solution—i.e., a solution that involved inhibiting the misleading heuristic. Prado and Noveck (2007) using a similar deductive reasoning task as the one we used provided convergent evidence that the resolution of such problems involved inhibitory control. In their study, participants were asked to determine whether a conditional rule such as “if there is not a B there is a triangle” was falsified (or verified) by an item (e.g., A and diamond). They reported increased activation in the right mid-dorsolateral prefrontal cortex (mid-DLPFC), the medial frontal areas (including the anterior cingulate area), the pre-supplementary motor area and the parietal cortices with increasing perceptual mismatch between the conditional rule and the item (i.e., when the perceptual matching bias was stronger). Critically, a psychophysiological interaction analysis revealed that the integration between the visual areas of the brain (supporting the perceptual matching heuristic) and mid-DLPFC decreased when the perceptual mismatch increased. Taken together the results suggest that overcoming the perceptual matching bias is rooted in part by the inhibitory control exerted by prefrontal regions (i.e., mid-DLPFC and the medial frontal cortex) on lower level visual regions.

Note that whereas the left lateral prefrontal structures (including the left IFG) supported the inhibition of the misleading heuristic during conditional reasoning in our studies (Houdé et al., 2000, 2001) subsequent studies reported activation in the right IFG (e.g., Noveck et al., 2004; Prado and Noveck, 2007; for reviews see Goel, 2007; Prado et al., 2011). We suspect that the activation in the left prefrontal areas of the brain reported in our seminal studies could be a consequence of the verbal nature of the executive training (given between the pre- and post-test) which would have favored using inhibitory control in verbal working memory after the training (i.e., during the post-test). This interpretation is coherent with previous studies showing that inhibition in verbal working memory is supported by the left prefrontal areas of the brain (Jonides et al., 1998).

The role of inhibitory control and the prefrontal cortex (including the inferior frontal gyrus, IFG) in deductive reasoning has been demonstrated not only using conditional reasoning but also syllogistic reasoning (De Neys and Van Gelder, 2009; Tsujii et al., 2010, 2011). For instance, Tsujii et al. (2010) investigated the network of brain areas involved in syllogistic reasoning. Critically, prefrontal regions including the right IFG—i.e., a region consistently activated when a prepotent response (or a heuristic) is inhibited (see Aron et al., 2004, 2014)—are specifically recruited when participants judge the validity of syllogisms in which the logical validity of the conclusion is in conflict with the belief of the participants (e.g., Valid incongruent syllogism: *No mammals are dogs/All German Shepherd are mammals/No German Shepherd are dogs*). Importantly, a follow-up study revealed that the ability to reason on belief laden syllogisms is impaired when the activity of the right IFG is disrupted using rTMS (i.e., repetitive Transcranial Magnetic Stimulation). This study provided additional evidence for a causal relation between the right IFG and the ability to overcome logical errors through the inhibition of heuristic thinking.

## Brain Imaging of Reasoning-Bias Inhibition in Children: The Example of Number Conservation

One of the most famous Piagetian problems used for testing reasoning in children is the number-conservation task (Piaget, 1983). In this problem, the child is first presented with two rows of tokens with the same number of tokens and the same length. After the child acknowledges that the two rows contain the same number of objects, the tokens in one of the rows are spread apart and the child is asked whether the two rows contain yet the same number of tokens. Children younger than 6 or 7 years of age tend to report that the longer row contains more tokens. According to Piaget (1952), young children make systematic errors in the number-conservation problem because they rely on an intuitive “illogical” mode of thinking which is a hallmark of the preoperational stage of cognitive development. When children reach 6 or 7 years of age, they successfully solve the number conservation task by understanding the reversibility of operations (any transformation can be cancelled out by the reverse transformation) which is evidence that children are in the concrete operational stage of development.

Following Piaget’s pioneer work, a growing number of studies were proposed to investigate the cognitive development of numeracy and raised numerous criticisms of Piaget’s theory. For instance, studies have demonstrated that newborns and infants understand that there is an invariance between number and physical transformations, even in contexts extremely similar as the one created in the number-conservation problem (Antell and Keating, 1983; see also Dehaene, 2011). A critical question for developmental psychologist is thus to understand why newborns and infants who have some knowledge of the relation between number and space will later on make systematic errors in the number-conservation problem until age 6 or 7. This non-linear pattern of development could be explained by the fact that children learn a number of heuristics during their childhood that are most of the times appropriate to find the solution except in context in which they are misleading and need to be inhibited (Houdé, 2000; Houdé and Borst, 2014). For instance, in Piaget’s number-conservation problem, children tend to rely on the misleading length-equals-number heuristic rather than on a counting or operational reversibility algorithm.

One of the challenges of today’s research in developmental psychology is thus to shift from the Piagetian (Piaget, 1983) and neo-Piagetian (see Demetriou, 1988 for a review) views that the conceptual change exclusively relies on the growing ability to coordinate multiple systems of operations to a view according to which conceptual change is in part rooted in a domain-general ability of selection-inhibition of competing strategies, i.e., heuristics (or intuitions) and logico-mathematical algorithms. Critically, at each age and in each situation the strengths of the heuristics and the algorithms fluctuate within a nonlinear dynamical system (Siegler, 1999; Houdé, 2000; Houdé and Borst, 2014). According to this new model, cognitive development occurs in bursts with sometimes errors occurring after success in both children and adults. This model is coherent with what we know of the structural changes of the brain from childhood to adulthood (Casey et al., 2005). Indeed, the inhibition of heuristics could remain challenging because the maturation of the prefrontal cortex sustaining inhibitory-control ability continues throughout childhood and adolescence.

To determine whether the growing ability to perform Piaget’s number-conservation problem is rooted in the growing ability to inhibit the *length-equals-number* heuristic due to the progressive maturation of the prefrontal cortex, we asked 60 children aged 5–10 to solve Piagetian problems in a functional magnetic resonance imaging (fMRI) study. We found that children who succeed in solving Piaget’s number-conservation problems (i.e., children aged 7 and older) recruited a parieto-frontal network including the right IFG and the bilateral intra parietal sulcus (IPS; Houdé et al., 2011)—two regions respectively involved in inhibition (e.g., Aron et al., 2004, 2014) and numeracy (e.g., Dehaene, 2011). In a subsequent fMRI study (Poirel et al., 2012), we provided evidence that the recruitment of the right IFG was directly related to the need to inhibit a heuristic by reporting a significant positive correlation between the BOLD (i.e., the blood-oxygen-level-dependent) signal in the rIFG and the inhibitory control efficiency as measured by an Animal Stroop task (Wright et al., 2003)—a Stroop task adapted for non-reading children. The results we garnered in schoolchildren are coherent with the ones we reported above in adolescents and adults for which failure to inhibit a heuristic led to systematic logical errors although they reached the formal operational stage according to Piaget’s theory. Note, however, that our developmental study on number conservation shows a right-inferior-frontal gyrus activation for inhibition (in line with Aron et al. (2004, 2014) meta-analysis reviews), while our adults study on deductive reasoning (Houdé et al., 2000) showed a left-inferior-frontal gyrus activation for inhibition. In this last study, there was no Stroop-correlation control, but the leftward lateralization was probably due to the strong verbal component (rules) of the logical task, involving self-regulatory inner speech. The number conservation problem is, inversely, a visuospatial task which fits well with a rightward lateralization of the activation.

## Conclusion

In this review we want to argue that learning to inhibit misleading heuristics from System 1 (i.e., intuitive system) when they interfere with the activation of the logical algorithms from System 2 (i.e., analytical system, see e.g., Evans, 2003; Kahneman, 2011) is the critical process that allows one to reason logically (Houdé, 2000; Goel, 2007; Prado and Noveck, 2007; De Neys and Van Gelder, 2009; Tsujii et al., 2010, 2011; Prado et al., 2011; Houdé and Borst, 2014). The new post-Piagetian theoretical framework we propose allows us to better understand why newborns and infants who possess an early ability to reason logically in different domains will later in life have the tendency to reason illogically. Typically, at all ages, overcoming systematic logical errors relies on blocking (i.e., inhibiting) our intuitions, a process that is highly dependent on the maturation of the prefrontal cortex (Borst et al., 2013). Finally, the ability to inhibit misleading heuristics remains challenging throughout our lifetime. Thus children, adolescents and adults may sometimes need “prefrontal pedagogy” to help them overcome their tendency to rely on intuitive heuristics and biases in reasoning tasks (Houdé, 2007).

## Conflict of Interest Statement

The authors declare that the research was conducted in the absence of any commercial or financial relationships that could be construed as a potential conflict of interest.
